# Effect of periodontal therapy on maxillary sinus mucous membrane thickening in chronic periodontitis: A split-mouth study

**DOI:** 10.15171/joddd.2018.026

**Published:** 2018-09-18

**Authors:** Vrushali N. Lathiya, Abhay P. Kolte, Rajashri A. Kolte, Dhawal R. Mody

**Affiliations:** Department of Periodontics and Implantology, VSPM Dental College and Research Centre, Nagpur, India

**Keywords:** Maxillary sinus, mucosal thickening, periodontitis, periodontal surgery

## Abstract

***Background.*** This study evaluated the effect of periodontal therapy on mucous membrane thickening in maxillary sinus in chronic periodontitis patients using radiovisiography (RVG) and cone-beam computed tomography (CBCT).

***Methods.*** The study population included 30 patients diagnosed with chronic periodontitis, exhibiting bilateral mucosal thickening of maxillary sinus. The selected sites were randomly assigned to group I (control group - not receiving periodontal therapy) and group II (test group - receiving periodontal therapy). The clinical parameters and mucosal thickening of the maxillary sinus were evaluated at baseline and after 9 months.

***Results.*** There was a significant decrease in the PPD, CAL as well as mucosal thickening in group II while, group I showed an increase in these parameters. In group II at the end of 9 months the mean mucosal thickening reduction as assessed by CBCT was 0.76±0.18, 0.73±0.24, 0.88±0.42 and 1.13±0.43 mm at the most anterior point (AP), the most posterior point (PP), the mid-point (MP), point of maximum thickness (MT) as well as in the length of the thickened mucosal lining, respectively.

***Conclusion.*** The results of our study indicated a reduction in the mucosal thickening of the maxillary sinus after surgical periodontal therapy. The trial was registered with the Clinical Trial Registry of India (Trial REF/ 2016/02/010805).

## Introduction

Maxillary sinuses are pneumatic cavities within maxillary bone. They communicate with the nasal cavity through the ostium and are lined with a thin respiratory mucous membrane referred to as the Schneiderian membrane which adheres firmly to the periosteum and is about 0.8‒1 mm in thickness.^1^ Normally, the sinus mucosa is not visualized on the radiograph. However, an infection or allergic process like chronic sinusitis might result in inflammation of the mucosa, making it visible on the radiograph. According to several reports, 10‒12% of the cases of maxillary sinusitis have been historically attributed to odontogenic infections.^2^ Conditions like periapical abscesses, periodontal diseases, dental traumas, tooth extractions or placement of dental implants violate the Schneiderian membrane and tend to increase the risk of maxillary sinusitis.^3^ Odontogenic maxillary sinusitis results from pathological interactions between neighboring periodontal and dental structures and maxillary sinus.

Periodontitis has been thought to be one of the potential causes of odontogenic maxillary sinusitis and subsequent thickening of the sinus mucosa is associated with it. Primarily, panoramic radiography, Water’s projection and intraoral radiography have been used in imaging of the maxillary sinus in dentistry.^4^ However, the complex anatomy of the oral and maxillofacial region makes it difficult to visualize important anatomical features due to the superimposition.^5^ Conventional radiographic techniques lack the accuracy to detect and measure the mucosal thickening of the maxillary sinus.

Computed tomography (CT), the gold standard for sinus diagnosis, provides multiple sections through the sinus at different planes allowing the visualization of hard and soft tissues. Recent introduction of cone-beam computed tomography (CBCT) for dental and maxillofacial imaging has facilitated the diagnosis of delicate structures in multiplanar reconstructions. With the advantage of lower radiation dose and an isotropic volume resolution CBCT can be used for imaging of paranasal sinuses.^6^

A fairly good amount of literature is available on the association between periodontitis and mucosal thickening of the maxillary sinus; however, to date, very few clinical studies have been carried out to identify the effect of periodontal therapy on maxillary sinus mucous membrane thickening. Therefore, the aim of the present study was to evaluate the effect of periodontal therapy on mucous membrane thickening in maxillary sinus in chronic periodontitis patients by radiovisiography (RVG) and CBCT.

## Methods

Thirty patients (13 males and 17 females) with chronic periodontitis and with a mean age of 45.86±5.84 years (range: 36–56 years) were recruited from those visiting the Department of Periodontics and Implantology, of our institute from July 2015 to December 2016. Radiographs were taken and only patients demonstrating bilateral mucosal thickening of the maxillary sinus were recruited.

Patients exhibiting generalized moderate-to-severe chronic periodontitis, as assessed by probing pocket depth (PPD) of ≥5 mm and clinical attachment level (CAL) of ≥5 mm and the presence of at least one of the first and second molars or second premolars in each left or right quadrants were included in the study. Patients were excluded if they presented with signs of acute non-odontogenic sinusitis, with a history of common cold and sinusitis in the past months, with a history of systemic diseases, with chronic or acute periapical lesions, with a history of oroantral fistulas, with pathologies of maxillary sinus, with allergies or drug use and with a history of periodontal treatment during the previous 6-month period.

The study protocol was approved by the Institutional Ethics Committee of VSPM Dental College and Research Centre, Nagpur; all the procedures coformed to the Helsinki Declaration of 1975, as revised in 2008. The study was registered with Clinical Trial Registry of India (Trial REF/ 2016/02/010805). The study details were explained to the participants individually and patients who volunteered to take part in the trial signed an informed consent form and were included. The selected sites were randomly assigned by a toss of coin to group I (control group - not receiving periodontal therapy) and group II (test group - receiving periodontal therapy).

For evaluation of oral hygiene and gingival health, plaque index (PI)^7^ and gingival index (GI)^8^ were obtained at baseline and at 9 months. Group I subjects were not subjected to any presurgical hygiene therapy and only group II patients were taken up for scaling and root planing. After the hygiene phase of therapy, PPD and CAL were recorded at baseline using custom-made occlusal acrylic stents with UNC – 15 color-coded periodontal probes (Hu-Friedy, Chicago, IL, USA). The patients were re-evaluated at 3 months to confirm the need for surgery. If the pockets remained unresolved (PPD: ≥5 mm) at the end of 3 months, the patients were taken up for surgery. Also, the mucosal thickening was measured by both RVG and CBCT at baseline and at 6 months after the surgical procedure. Patients in the control group were taken up for periodontal therapy after the study. All the clinical periodontal parameters were recorded by a single operator (VL) who was masked to the treatment status of the sites.

### Clinical procedure

Only the group II patients were subjected to surgical periodontal therapy. Under local anesthesia, a mucoperiosteal flap was reflected to gain access to the underlying tissues and thorough debridement was carried out. Granulation tissue was removed, and the exposed root surface was scaled and planed using hand and ultrasonic instruments. The flaps were then secured with 3-0 Mersilk sutures (Alsilk, Aalay Surgicals Pvt. Ltd., India) using interrupted suturing technique and periodontal dressing was placed. The patients were prescribed analgesics for a period of one week and 0.2% chlorhexidine mouthrinse for 2 weeks post-surgically. All the surgeries were performed by a single operator (AK).

### Assessment of mucosal thickening

IOPA radiograph with paralleling technique with film holder (XCP Rinn) was taken using RVG and the linear measurements were carried out at baseline and 6 months after surgery to measure the thickness of the mucosal lining of the maxillary sinus using a software program (DIGORA TM OPTIME RVG Software, Finland). CBCT (KODAK 9000C 3D Extraoral Imaging System, Carestream Health, Inc, France) images were taken and commercially available CBCT software was used for the image analysis. The panoramic and cross-sectional views of the maxilla were reconstructed for evaluations and measurements.

During both RVG and CBCT measurements the floor of the maxillary sinus was traced in the area of mucosal thickness and this determined the total length of the thickened mucosa. Mucosal thickening was considered to be present when the thickness of the sinus mucosa was ≥1 mm. The thickness was measured in millimeters from the floor of the sinus to the highest border of the mucosa.

The following measurements were made:

1. Thickness of the mucosal lining at the most anterior point (AP).
2. Thickness of the mucosal lining at the most posterior point (PP).
3. Thickness of the mucosal lining at a point midway between the most anterior and the most posterior points (MP).
4. Thickness of the mucosal lining at the point of maximum thickness of the sinus mucosa.

All the radiographic measurements were made by a single operator (RK) who was blinded to the clinical measurements and the treatment status of the sites.

### Statistical analysis

Data were analyzed using a statistical software program (STATA Corp. 2013. Stata Statistical Software: Release 13. College Station, TX: Stata Corp LP). Statistical significance was set at P<0.05. Continuous variables (age, PI, GI, PPD, CAL, recession, mucosal thickness) were presented as mean ± SD. PI, GI, PPD, CAL and mucosal thickening were compared at different time intervals in each group using paired t-test. Mean changes in these study parameters were compared between group I and group II by Wilcoxon rank sum test (Mann-Whitney test). RVG and CBCT analysis of mucosal thickening was carried out at different time intervals in each group by performing paired t-test. Mean changes in RVG and CBCT analysis of mucosal thickening were compared between groups I and II by performing Wilcoxon rank sum test (Mann-Whitney test). Mucosal thickening in terms of age and PPD was compared in each group using independent t-test.

## Results

In general, patients exhibited good oral hygiene throughout the duration of the study. Baseline full-mouth plaque score was 3.01±0.28, while at 9 months (6 months after the surgery), it decreased to 1.22±0.50. The mean GI decreased from 1.46±0.25 at baseline to 1.04±0.12 at 9 months. PI and GI scores, when compared with baseline to that at the end of 9 months, showed statistically significant decrease (P<0.001).

The PPD and CAL also exhibited a significant change at the end of 9 months compared to baseline. In group I there was an increase in the mean PPD and CAL compared to baseline. Group II showed a statistically significant reduction in PPD and CAL gain at the end of 9 months. When the mean PPD and CAL changes in group I were compared with those in group II, the difference was statistically significant (P<0.0001) (Table 1).

**Table 1:** Measurements of clinical parameters (mean ± SD^†^) at baseline and at 9 months (intra-group comparison)

**Parameters**	**Group I**			**Group II**		
	** Baseline**	**9 months**	**P-value**	** Baseline**	** 9 months**	**P-value**
**PPD (mm)**	5.23±0.14	5.26±0.20	<0.0001*	5.29±0.39	3.05±0.50	<0.0001*
**CAL (mm)**	5.52±0.30	5.65±0.30	<0.0001*	5.59±0.36	3.97±0.46	<0.0001*

^*^highly significant ^†^SD = standard deviation

### Assessment of mucosal thickening of maxillary sinus by RVG and CBCT

#### RVG analysis of mucosal thickening

The mean mucosal thickness at AP, PP, MP, at the point of maximum thickness as well as the length of the mucosal thickening in group I (control) and group II (test) at baseline and at the end of 9 months are presented in Table 2. Group I showed a mean increase in the mucosal thickening at the end of 9 months. The increase in thickening was highly significant at AP, PP and MP (P<0.0001) while it was non-significant at the point of maximum thickness (P=0.2680). At the end of 9 months the thickened sinus mucosa also exhibited a statistically significant increase as compared to baseline (P=0.0403).

**Table 2:** Measurements of mucosal thickening by radiovisiography (RVG) (mean ± SD^†^) at baseline and at 9 months (intra-group comparison)

**Mucosal thickening (mm)**	**Group I**			**Group II**		
	**Baseline**	**9 months**	**P-value**	**Baseline**	**9 months**	**P-value**
**AP**	1.80±0.26	1.87±0.25	<0.0001^*^	1.7±0.33	1.00±0.18	<0.0001^*^
**PP**	1.91±0.33	2.01±0.33	<0.0001^*^	1.64±0.35	1.00±0.21	<0.0001^*^
**MP**	2.24±0.40	2.32±0.39	<0.0001^*^	2.24±0.50	1.32±0.28	<0.0001^*^
**Point of maximum thickness**	2.84±0.40	2.91±0.49	0.2680^‡^	2.91±0.52	1.89±0.42	<0.0001^*^
**Length of thickened band**	29.55±2.64	29.63±2.63	0.0410^§^	29.28±2.61	29.18±2.64	0.14^‡^

^*^highly significant ^†^SD = standard deviation ^‡^non-significant ^§^P<0.05, significant

On the other hand, group II showed a decrease in the mean mucosal thickening at the end of 9 months, which was statistically significant at all the points (P<0.0001) except for the length of the thickened mucosa, which was not significant (P=0.1410).

#### CBCT analysis of mucosal thickening

**Figure 1:**
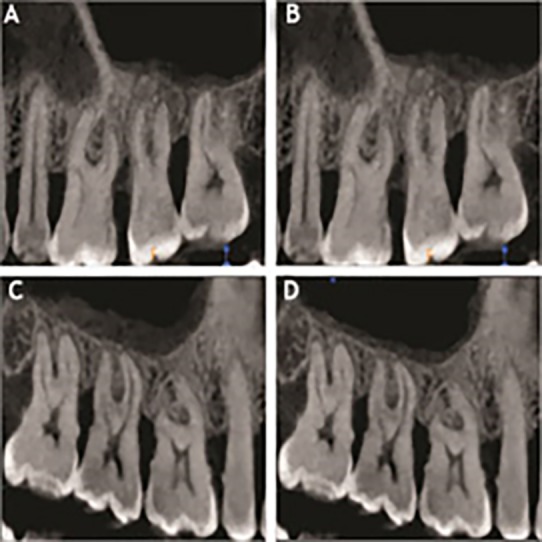
(A) CBCT analysis of mucosal thickening for group I at baseline. (B) CBCT analysis of mucosal thickening for group I at the end of 9 months. (C) CBCT analysis of mucosal thickening for group II at baseline. (D) CBCT analysis of mucosal thickening for group II at the end of 9 months.

The CBCT measurements also followed a similar trend as depicted by the RVG. Group I exhibited an increase in the mucosal thickness at the end of 9 months while group II showed a reduction in the mucosal thickening of the maxillary sinus (Table 3) (Figure 1). For group I, there was an increase in the thickness of the sinus mucosa at all the points at the end of 9 months except for the AP, where a reduction in the thickening of the sinus mucosa was observed. However, it was not significant statistically at any point, except for the length of the thickened sinus mucosa, which showed a statistically significant increase as compared to baseline (P=0.0009). In group II at the end of 9 months the mean mucosal thickening reduction was highly significant at all the points (P<0.0001) and statistically significant for the length of the thickened mucosa (P=0.0081).

**Table 3:** Measurements of mucosal thickening by CBCT (mean ± SD^†^) at baseline and at 9 months (intra-group comparison)

**Mucosal thickening (mm)**	**Group I**			**Group II**		
	**Baseline**	**9 months**	**P-value**	**Baseline**	**9 months**	**P-value**
**AP**	1.99±0.29	1.96±0.25	0.3178^‡^	1.94±0.38	1.18±0.26	<0.0001^*^
**PP**	2.09±0.34	2.13±0.23	0.1820^‡^	1.92±0.37	1.19±0.24	<0.0001^*^
**MP**	2.53±0.53	2.6±0.42	0.1824^‡^	2.48±0.49	1.6±0.37	<0.0001^*^
**Point of maximum thickness**	3.09±0.46	3.15±0.44	0.0974^‡^	3.23±0.51	2.09±0.45	<0.0001^*^
**Length of thickened band**	29.54±2.60	29.84±2.60	0.0009^§^	29.54±2.60	29.47±2.63	0.0081^§^

^*^highly significant ^†^SD = standard deviation ^§^P<0.05, significant

When the baseline versus 9 months change in the mucosal thickening was compared between group I and group II it was highly significant at all the points as well as in the length of the thickened mucosal lining both in RVG as well as CBCT Evaluations (Table 4).

**Table 4:** Measurements of radiographic parameters (mean ± SD^†^) at baseline and at 9 months (inter-group comparison)

**Mucosal thickening** **(mm)**	**RVG**			**CBCT**		
	**Group I**	**Group II**	**P-value**	**Group I**	**Group II**	**P-value**
**AP**	-0.063±0.07	0.69±0.23	<0.001^*^	0.026±0.14	0.76±0.18	<0.001^*^
**PP**	-0.093±0.12	0.64±0.28	<0.001^*^	-0.043±0.17	0.73±0.24	<0.001^*^
**MP**	-0.08±0.13	0.91±0.30	<0.001^*^	-0.063±0.25	0.88±0.42	<0.001^*^
**Point of maximum thickness**	-0.07±0.34	1.01±0.44	<0.001^*^	-0.063±0.20	1.13±0.43	<0.001^*^
**Length of thickened band**	-0.08±0.20	0.096±0.35	<0.0037^§^	-0.076±0.11	0.073±0.14	<0.001^*^

^*^highly significant ^†^SD = standard deviation ^§^P<0.05, significant

### Comparison of RVG and CBCT analysis of mucosal thickening

A variation was observed in the measurements of the mucosal thickening of maxillary sinus by RVG and CBCT in both groups at baseline and at the end of 9 months (Table 5).

**Table 5:** Comparison of CBCT and RVG analysis of mucosal thickening (in mm) in 2 groups at different time intervals

Parameter	Time	Group I		P-value	Group II		P-value
		RVG	CBCT		RVG	CBCT	
AP	Baseline	1.80±0.26	1.99±0.29	0.0139^§^	1.70±0.33	1.94±0.38	0.0139^§^
	9 months	1.87±0.25	1.96±0.24	<0.0001^*^	1.00±0.18	1.18±0.26	<0.0001^*^
PP	Baseline	1.91±0.32	2.09±0.34	0.0518^‡^	1.64±0.35	1.92±0.37	0.0518^‡^
	9 months	2.01±0.33	2.13±0.23	0.0002^§^	1.00±0.20	1.19±0.23	0.0002^§^
MP	Baseline	2.24±0.40	2.53±0.53	0.0189^§^	2.24±0.50	2.48±0.49	0.0189^§^
	9 months	2.32±0.39	2.6±0.42	<0.0001^*^	1.32±0.28	1.6±0.37	<0.0001^*^
Point of Maximum Thickness	Baseline	2.84±0.40	3.09±0.46	0.3000^‡^	2.91±0.52	3.23±0.51	0.3000^‡^
	9 months	2.91±0.49	3.15±0.44	<0.0001^*^	1.89±0.42	2.09±0.45	<0.0001^*^
Length of thickened band	Baseline	29.55±2.64	29.77±2.64	0.7534^‡^	29.28±2.61	29.54±2.60	0.7534^‡^
	9 months	29.63±2.63	29.84±2.60	<0.0001^*^	29.18±2.64	29.47±2.63	<0.0001^*^

^*^highly significant ^†^SD = standard deviation ^‡^non significant ^§^P<0.05, significant

In group I, the difference in the values for AP and MP were statistically significant at baseline and highly significant at 9 months (P<0.0001). However, at the PP, point of maximum thickness and the length of the thickened mucosal lining, the difference in the RVG and CBCT values was non-significant at baseline but statistically significant at the end of 9 months.

In group II, the difference in the values for AP and MP were significant at baseline and highly significant at 9 months. However, at the PP point of maximum thickness and the length of the thickened mucosal lining, the difference in the RVG and CBCT values was statistically non-significant at baseline but highly significant at the end of 9 months.

## Discussion

In the present study, we evaluated the effect of periodontal therapy on the mucosal thickening of maxillary sinus. Strict inclusion criteria were employed to select only generalized chronic periodontitis patients with mucosal thickening of maxillary sinus, while excluding patients who demonstrated any signs of acute non-odontogenic sinusitis, history of common cold and sinusitis in the past 3 months, systemic diseases, allergies or drug use and those who had undergone periodontal treatment in previous 6 months. We also aimed to ensure that if resolution of mucosal thickening was observed, it was due to the periodontal treatment rendered and not medications taken by the subjects.

In present study, in order to evaluate the effect of periodontal therapy on the mucous membrane thickening of maxillary sinus, group I subjects were not given any periodontal therapy while group II subjects were treated surgically. Surgical periodontal therapy was selected over non-surgical periodontal therapy in our study because non-surgical periodontal therapy has a limitation in improving periodontal status beyond 4.2 mm as shown by the concept of critical probing depth.^10^ The greater the depth of periodontal pockets, the lower the efficacy of non-surgical periodontal therapy, while surgical periodontal therapy has been reported to result in a greater pocket depth reduction and gain in CAL. There was a reduction in the mean PPD and gain in CAL in group II, while group I showed an increase in mean PPD and also CAL loss when compared to baseline.

Radiographic examination is routinely used for the visualization of maxillary sinus and is a critically important tool for detection of sinus pathology. Numerous 2D and 3D imaging modalities like panoramic radiography, Water’s projection, intraoral radiography and CT as well as CBCT have been used for the imaging of maxillary sinus.^11^ At the time this study was undertaken, no CBCT-based evidence was available on the resolution of maxillary sinus mucosal thickening in response to periodontal treatment of maxillary teeth in patients with chronic periodontitis. In this study, we used RVG as well as CBCT to assess the thickness of the mucosal lining of the maxillary sinus.

The normal thickness of the maxillary sinus mucosa, also known as the Schneiderian membrane, is reported to be 0.8‒1 mm while a thickness of >1 mm was considered as evidence of mucosal thickening (MT). Phothikhun et al^12^ and Shekhi et al^13^ defined the MT when it was >1 mm and Shahidi et al^14^ considered a mucosal thickening of >1 mm as pathological. On the contrary, Janner et al^15^ used CBCT to evaluate sinus mucosal thickness among dental patients and a mucosal thickness of >2 mm was used as a cut-off for mucosal thickening. Similarly, Maillet et al^16^ and Lu et al^17^ considered a mucosal thickening of >2 mm as pathological. Soikkonen and Ainamo^18^ considered the presence of diffuse radiopacities along the margins of the sinus without well-defined rounded outlines as a criterion for maxillary sinus mucosal thickening. However, the above-mentioned study used panoramic radiographs which do not allow accurate measurements of sinus mucosal thickness.

Considering the inconsistencies and for a detailed and more accurate understanding, the mucosal thickness of the maxillary sinus was assessed at four different points: at AP (in mm), at the PP (in mm), MP (in mm) and at the point of maximum thickness of the sinus mucosa (in mm). Also, the total length of the thickened band was measured.

A reduction in the thickness of the mucosal lining of the maxillary sinus was seen in group II when it was subjected to periodontal therapy. On the other hand, no reduction was seen in the thickness of the mucosal lining in group I which was deprived of any sort of periodontal therapy; rather, it showed a slight increase in the thickness of the sinus mucosa at the end of 9 months when compared to baseline. It can be inferred that the treatment of periodontal disease to reduce the pathogens and pathogenic products results in a decrease in mucosal thickness.

The possible mechanism for the thickening of sinus mucosa is the presence of deep periodontal pockets which evoke a local reaction in the sinus mucosa, such as edema, round cell infiltration, fibrosis or cystic degeneration. This response also occurs in cases with a fairly thick layer of bone separating the apical area of the infected tooth from the maxillary sinus.^19^ Maxillary sinusitis as a result of periodontal infections can be caused either by the spread of microorganisms and their products as well as the cytokines via the numerous anastomoses between the blood and lymph vessels in the apical region of the tooth and the corresponding vessels in the sinus mucosa or by a direct spread of infection through the porous maxillary bone and the supporting tissues.

These findings are consistent with those reported by Falk et al^20^ and Engstrom et al,^21^ who reported a resolution in maxillary mucosal thickening in response to a successful periodontal therapy. The above-mentioned studies reported a complete resolution of the sinus mucosal thickening. On the contrary, a partial resolution of the mucosal thickening was observed in our study. This variability in the results can be attributed to differences in imaging modalities that were used. The detection of sinus mucosal thickening using CBCT in this study was expected to be more sensitive than conventional radiography as used in reference studies. With less sensitive imaging, partially resolved mucosal thickening of maxillary sinus might appear as fully resolved, resulting in an increased incidence of reported full resolution.

In the present study, we additionally compared the assessment of mucosal thickening of the maxillary sinus by RVG to CBCT. A difference in the assessment of sinus mucosal thickening was found throughout as reflected by the variation in the values of mucosal thickening when evaluated by RVG and CBCT. Interpretation of plain radiographs can vary widely among different observers, and there is a high rate of false negative results.^22^ Because an unresolved sinusitis might be exacerbated by an untreated dental condition, having both axial and coronal views as provided by CBCT allows the clinician to assess the relationship of a periapical lesion or periodontal infection to a sinus floor defect and any resultant changes in the soft tissue of the sinus.^23^ This finding is supported by Cymerman et al,^24^ who found that thickening of the sinus membrane was identified four times more often by CBCT than with conventional periapical radiographs and that it was useful for differentiating the etiology and extension of oral pathology with respect to maxillary sinus.

Thus, it can be naturally commented that periodontal disease is a potential cause of sinus mucosal thickening and when successful periodontal therapy is provided as indicated by the reduction in PPD and CAL gain, it leads to a reduction in the mucosal thickening of the maxillary sinus. Also, CBCT can be considered as a standard in assessing these sinus pathologies. This study has several clinical implications as well. Periodontal infection is an etiologic factor for maxillary sinusitis which is the major cause of thickening in symptomatic individuals. Mucosal thickening of maxillary sinus and other sinus pathologies might pose problems when a sinus augmentation surgery is planned. Thus, periodontal treatment should be performed before sinus augmentation surgery to reduce the inflammation of the sinus mucosa.

## Conclusion

To the best of our knowledge, currently there are no guidelines or consensus on the classification and management of mucosal abnormalities before sinus augmentation surgery. The present study proposed a innovative and detailed assessment methodology for mucosal thickening of the maxillary sinus. The results indicated that the surgical periodontal therapy led to a reduction in microbial load and their pathogenic products, which ultimately affected the mucosal thickening, leading to its resolution. A larger sample size is needed to determine the stability of the results and to improve the radiographic assessment of the results. Although strict inclusion and exclusion criteria were employed to include only generalized chronic periodontitis, the possibility remains that the mucosal thickening in some of the patients might have resulted from or lingered because of etiologies other than chronic periodontitis. Therefore within the limits of present study, it can be concluded that both RVG and CBCT demonstrated a reduction in the mucosal thickening of the maxillary sinus after surgical periodontal therapy. Although both RVG and CBCT can be used to assess the mucosal thickening of the maxillary sinus, there was a variation in the measurements and CBCT can be relied upon for precise measurements.

## Acknowledgments

The authors would like to acknowledge the staff of the Department of Periodontics and Implantology, VSPM Dental College and Research Centre, Nagpur for their support.

## Authors’ contributions

VNL contributed to definition of intellectual content, literature search, clinical studies, experimental studies and data acquisition. APK contributed to the concept and design of the study, literature search, data acquisition, data analysis, manuscript preparation and editing. RAK contributed to the design, definition of intellectual content, literature search, clinical studies, data acquisition, data analysis, manuscript preparation as well as editing. DRM contributed to definition of intellectual content, literature search and manuscript review. All the authors have read and approved the final manuscript.

## Funding

Not applicable.

## Competing interests

The authors declare no competing interests with regards to the authorship and/or publication of this article.

## Ethics approval

The study was approved by the Institutional Ethics Committee of VSPM Dental College and Research Centre under the code IEC/VSPMDCRC/21/2015.
